# A bibliometric study on mathematical oncology: interdisciplinarity, internationality, collaboration and trending topics

**DOI:** 10.1007/s11538-025-01544-9

**Published:** 2025-11-04

**Authors:** Kira Pugh, Linnéa Gyllingberg, Stanislav Stratiev, Sara Hamis

**Affiliations:** 1 Department of Information Technology, Uppsala University, Uppsala, Sweden; 2 Department of Mathematics, University of California Los Angeles, Los Angeles, CA USA; 3 Northell, Birmingham, UK

**Keywords:** Mathematical cancer research, Mathematical biology, Bibliometric analysis, Science of science, Scientometrics

## Abstract

Mathematical oncology is an interdisciplinary research field where the mathematical sciences meet cancer research. Being situated at the intersection of these two fields makes mathematical oncology highly dynamic, as practicing researchers are incentivised to quickly adapt to both technical and medical research advances. Determining the scope of mathematical oncology is therefore not straightforward; however, it is important for purposes related to funding allocation, education, scientific communication, and community organisation. To address this issue, we here conduct a bibliometric analysis of mathematical oncology. We compare our results to the broader field of mathematical biology, and position our findings within theoretical science-of-science frameworks. Based on article metadata and citation flows, our results provide evidence that mathematical oncology has undergone a significant evolution since the 1960s marked by increased interactions with other disciplines, geographical expansion, larger research teams, and greater diversity in studied topics. The latter finding contributes to the greater discussion on which models different research communities consider to be valuable in the era of big data and machine learning. Further, the results presented in this study quantitatively motivate that international collaboration networks should be supported to enable new countries to enter and remain in the field, and that mathematical oncology benefits both mathematics and the life sciences.

**Supplementary Information:**

The online version contains supplementary material available at 10.1007/s11538-025-01544-9.

## Introduction

Mathematical oncology is an interdisciplinary research field in which mathematical modelling, analysis, and simulations are used to study cancer (Altrock et al. 2015). Since the term *mathematical oncology* was introduced in the literature in the early 2000s (Gatenby and Maini 2003), the application areas of mathematics in cancer research have increased with the recent surge in available cancer data ranging from omics data on the gene expression level (Vandereyken et al. 2023), to imaging data on the cell and tumour levels (Bond et al. 2022; Rong and Liu 2024), biomarker data on the individual patient level (Passaro et al. 2024), and large-scale register data on the human population level (Tucker et al. 2019).

Therefore, although mathematical oncology is not a new research field, with early models of tumour growth in mice published in the 1930s (Mayneord 1932), it is dynamic. The field’s objective to study one of our biggest health threats – cancer – incentivises researchers to quickly adapt to advances pertaining to new cancer data, therapies, and clinical practices. Such advances have the ability to rapidly alter global research directions, as opposed to mathematical research advances that promote methodological revolutions that slowly change scientific practices (Krauss 2024). The fact that mathematical oncology is motivated by cancer research, yet grounded in mathematics, provides a tangible example of how research fields, particularly interdisciplinary ones, are not rigidly defined (McGillivray et al. 2022). Rather, they change over time which complicates the practice of clearly defining the delimiters that describe what a specific research field is, and what it is not.

The ability to quantitatively understand and describe a research field is helpful for actors operating both within and outside the field, including researchers, students, policy-makers, stakeholders, and the general public. Importantly, such an understanding can influence the design of university and school curricula, resource allocation within funding bodies and institutions, and science communication strategies.

One effective approach to quantitatively study research fields is through bibliometric analysis, where statistical methods are used to analyse patterns in science communication data. Bibliometric studies enable large-scale quantitative mappings of research fields, including analysis of historical, current, and emerging research trends (Donthu et al. 2021; de Oliveira et al. 2019). Platforms that host large databases of scientific publication data, such as Web of Science (Clarivate 2025), Scopus (Elsevier 2025), and OpenAlex (OurResearch 2022), facilitate bibliometric data collection. Coupled with the uptick in user-friendly bibliometric visualisation tools, including VOSviewer (Van Eck and Waltman 2009), biblioshiny (Aria and Cuccurullo 2017), CiteSpace (Chen 2006), and CitNetExplorer (Van Eck and Waltman 2014), it is becoming increasingly accessible to perform bibliometric studies, as is reflected by their rapid growth in numbers (Cheng 2024).

Today, it is common practice for research councils and institutes to perform bibliometric data analysis to evaluate the impact of published works produced by specified researchers and projects (Thelwall et al. 2023). Others who conduct bibliometric work include both science-of-science researchers, potentially “looking in” on a research field from the outside, and practitioners within the studied field. When coupling domain knowledge with theoretical frameworks from science-of-science, a broad range of topics that are traditionally studied qualitatively can be quantitatively assessed through bibliometric analysis (Lund 2021). These topics include research ethics (Gureyev and Mazov 2022), values (Birhane et al. 2022), and gender equality (Brück 2023).

Of relevance to this article, previous bibliometric studies have analysed the intersection of cancer research and disciplines that neighbour mathematics such as artificial intelligence (Koçak and Akçali 2024), machine learning (Lin et al. 2023), and deep learning (Wang et al. 2024). These studies largely focus on comparing publication and citation activity between countries and institutions, and highlight the rise of machine learning methods following the last decade’s string of machine learning breakthroughs that received attention from both the scientific and broader community.

In this work, we contribute a bibliometric study on the intersection of cancer research and mathematics, *i.e.,* *mathematical oncology*. By analysing article metadata, we investigate interdisciplinarity, internationality, and collaboration patterns, as well as trending research topics in the field, between the years 1961 and 2024. We quantitatively compare our results to trends in the more general field *mathematical biology*, and position our findings within theoretical frameworks rooted in science-of-science (Fortunato et al. 2018) and culture of science (Franklin 1995).

## Methods

### Study aims

The design of a bibliometric study should be tailored to its aims (Öztürk et al. 2024). In this study, our aim is to answer the following core research questions on interdisciplinarity, internationality, collaboration, and trending topics, respectively:

1. Which disciplines have influenced, and are influenced by, mathematical oncology? In other words, who is mathematical oncology for?
2. How has mathematical oncology expanded globally over time? Do new countries enter the field independently or through connections with previously engaged countries?
3. How collaborative is mathematical oncology in comparison to other fields? Has this changed over time?
4. How has mathematical oncology adapted to wider research breakthroughs? Has the research focus of mathematical oncology changed over time?

### Two-stage bibliometric methodology

To study research questions R1 to R4, and the development of mathematical oncology within its disciplinary context as a subfield of mathematical biology, we adopt a journal-first approach that follows a two-stage bibliometric methodology (Waltman and van Eck 2013). In the first stage, we perform a data-driven selection of focus journals and, in the second stage, we classify articles from the these as belonging to mathematical oncology or not.

While a direct keyword-based article selection method (that bypasses the journal filter) would yield a larger dataset by capturing all articles that mention some specified cancer and mathematics keywords, it would also introduce significant keyword bias and noise. Such unfiltered methods often include articles that reference keywords superficially, without necessarily engaging in the epistemic practices of the field studied under the bibliometric lens (Chen and Xiao 2016). Via our two-stage process, we are instead able to focus on core mathematical biology journals, ensuring that the articles analysed are, in fact, grounded in the field of mathematical biology. This approach also provides a built-in reference set in the form of non-oncology articles from the same journals, allowing for direct comparison between mathematical oncology and its parent field mathematical biology after ensuring sufficient data – articles – in both groups (Rogers et al. 2020).

It is important to note that our journal-first approach excludes all mathematical oncology work published outside our focus journals, which represents a substantial portion of the field. As the field has increasingly integrated with practical, medical, and industrial applications, mathematical oncology articles are now more frequently published in multidisciplinary journals, such as Proceedings of the National Academy of Sciences (PNAS), life science journals like Nature Communications, and specialised cancer journals such as Cancer Research.

### Journal selection

**Fig. 1:**
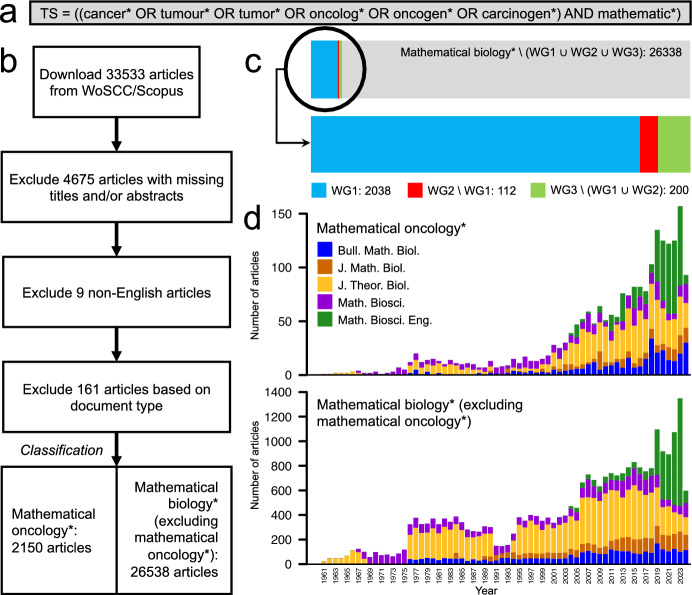
**Article selection and classification.** (a) The topic search (TS) query used in the WoSCC to identify mathematical oncology articles and inform the journal selection. (b) The data collection, cleaning and classification procedure. (c) The proportion of candidate keyword groups (WG) 1-3 proposed in the article classification, with article counts given after the colons. (d) Annual article counts for the focus journals following the article classification in (b)

In our journal-first approach, our first task is to identify mathematical biology journals that frequently publish mathematical oncology articles. To identify these journals, we search the Web of Science Core Collection (WoSCC) for articles containing specific keywords: both a cancer-related term and a term containing the substring mathematic* (Figure 1a). We use the WoSCC as our main database as it has excellent coverage of the mathematical biology literature (Supplementary Material, S3).

For all journals that have published more than 100 articles that match the keywords, we evaluate their relevance to mathematical biology in a twofold manner. First by identifying their journal classification according to the Quacquarelli Symonds (QS) scheme (2025), and second by examining their self-authored aims and scopes as written on the journal websites (Supplementary Material, S1). Our data-driven assessment reveals five mathematical biology journals with more than 100 mathematical oncology articles. We will refer to these as our *focus journals* throughout this study, and they are: the Bulletin of Mathematical Biology (BMB), the Journal of Mathematical Biology (JMB), the Journal of Theoretical Biology (JTB), Mathematical Biosciences (MB), and Mathematical Biosciences and Engineering (MBE), as listed in Table 1.

**Table 1:** **The mathematical biology focus journals researched in this study**. The Bulletin of Mathematical Biology was originally established in 1939 under the name Bulletin of Mathematical Biophysics

Journal name (ISO4)	Established (first mathematical oncology article)
Bulletin of Mathematical Biology (Bull. Math. Biol.)	1972 (Swan (1976))
Journal of Mathematical Biology (J. Math. Biol.)	1974 (Merrill (1984))
Journal of Theoretical Biology (J. Theor. Biol.)	1961 (Gause (1961))
Mathematical Biosciences (Math. Biosci)	1967 (Bellman et al. (1967))
Mathematical Biosciences and Engineering (Math. Biosci. Eng.)	2004 (Banks et al. (2004))

### Data collection

We collect article metadata from the WoSCC using a WoS API (Clarivate 2025). Specifically, we download metadata for all articles published in our focus journals up to and including the year 2024. For each article, we obtain the title, publication year, author names and affiliations, abstract, author keywords, references, and a list of items that cite the article. Articles published in MB between 1967 and 1975 are not indexed in the WoSCC, thus we download their metadata from Scopus.

### Data cleaning and article classification

Before proceeding with our bibliometric analysis, we clean the downloaded metadata in three steps: first, we remove articles with missing titles and/or abstracts; next, we omit non-English articles; and finally, we exclude documents classified as editorial material, notes, corrections, letters, biographical-items, and reprints 1b. As a result, we end up with only original research and review papers.

After data cleaning, we classify each article as belonging to one of two datasets: mathematical oncology* or mathematical biology* (excluding mathematical oncology*). Throughout this article, we will use asterisks to denote these datasets (as opposed to the full research fields). To classify which of the two datasets an article belongs to, we use keyword matches in the article’s title, abstract, and author keywords. If the article contains at least one cancer-class-keyword match, it belongs to the mathematical oncology* dataset and, otherwise, it does not.

It is not trivial to determine which keywords to include in bibliometric article classifications, as too narrow keywords might exclude articles, and too broad keywords might over-include articles in some specified class (Chen and Xiao 2016). To approach this problem for our specific dataset, we formulate three candidate keyword groups (WGs) where WG1 includes the general cancer terms listed in Figure 1a; WG2 includes a list of 57 cancer types such as leukemia, melanoma, and glioma; and WG3 includes a list of 19 words that are commonly mentioned alongside cancer such as chemotherapy and radiotherapy. A full list of keywords for all WGs is available in the Supplementary Material (S4). The frequency of articles that contain keywords in the studied WGs is shown in Figure 1c, and the resulting annual dataset sizes, after data cleaning and the final article classification, are shown in Figure 1d.

To determine which WGs to include in our classification, we evaluate their predictive performance manually by reading a subset of randomly selected article abstracts from each WG and, when needed, the full manuscripts. For WG1, WG2$\backslash$WG1, and WG3$\backslash$(WG1$\cup$WG2), we examine 100 articles predicted to be in mathematical oncology*, and 100 articles predicted to be in mathematical biology* (excluding mathematical oncology*). In brief, we found that using WG1$\cup$WG2 yielded the best performance – neither over-excluding nor over-including articles in the mathematical oncology* dataset. Accordingly, we classify any article containing at least one keyword from WG1$\cup$WG2 in the title and/or abstract as a mathematical oncology* article.

Leading up to this chosen classification, our evaluation showed that using WG1 as a classifier achieves 99% precision, i.e., 99% of the articles predicted as mathematical oncology* were also classified as such by human reviewers, but misses multiple relevant articles from WG2$\backslash$WG1. In contrast, WG3$\backslash$(WG1$\cup$WG2) achieves a 96% false discovery rate, i.e., 96% of articles predicted as mathematical oncology* were not classified as such by humans, and is consequently excluded from the classification keywords. Detailed quantitative results from the keyword classification evaluations are presented in a confusion matrix in the Supplementary Material (S4).

### Journal classification for citation analysis

To analyse citation flows, we categorise citing journals into seven disciplinary areas of interest. Beyond our focus journals, these are: mathematics and life sciences, mathematics, life sciences, multidisciplinary, STEM and life sciences, and other. To enable this categorisation to be fine-grained in subject areas close to mathematical oncology, and coarse-grained elsewhere, we customise a journal classification that builds on the well-established QS subject rankings (2025) and the All Science Journal Classification (ASJC) scheme (Elsevier 2025). In the ASJC scheme, a journal is assigned one or more category codes based on journal metadata, and the QS subject rankings further aggregate these codes into subject and broader faculty areas. Here, we use ASJC-QS classifications to directly map each citing journal to one of our seven disciplinary categories (Supplementary Material, S2).

### Data visualisation

We use R (R Core Team 2025) and the R-package biblioshiny (Aria and Cuccurullo 2017) to visualise bibliometric data. Instructions on how to generate the figures presented in this article are available on the study’s GitHub repository.

## Results

### On interdisciplinarity in mathematical oncology

As a first research question (R1) we ask: *Which disciplines influence and are influenced by mathematical oncology?* Equivalently put, is mathematical oncology a research field for (a) mathematicians, (b) cancer researchers, (c) mathematical biologists, (d) others, or (e) all of the above? Here, we take a discipline-centered bibliometric approach to answer this question.

**Fig. 2:**
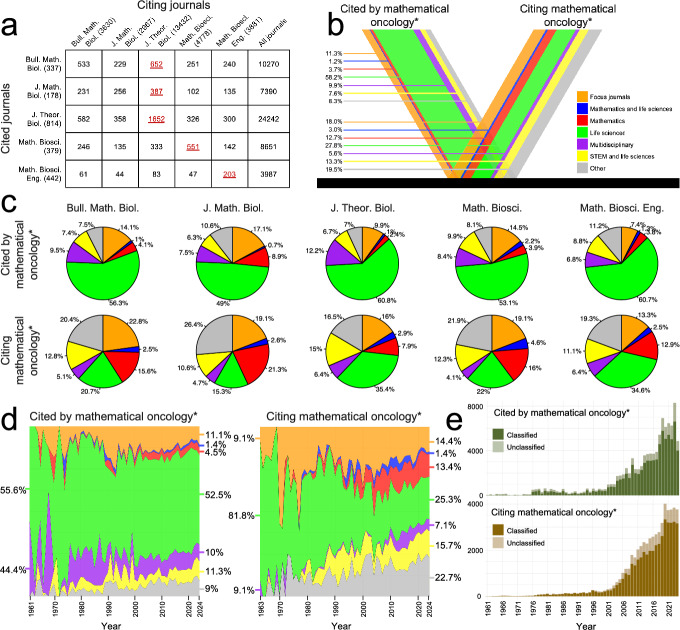
**Interdisciplinarity in mathematical oncology.** (a) The citation matrix shows how often mathematical oncology* articles (rows) are cited by mathematical biology* articles (columns). The right-most column shows the number of row-wise citations from all journals (including those beyond our focus journals). The total number of mathematical oncology* articles per journal are shown in row-legend parentheses, and the total number of mathematical biology* articles per journal are shown in column-legend parentheses. The maximum number of citations per row are red and underlined. (b) The citation-V shows the percentage of articles in different disciplinary categories that are cited by mathematical oncology* (left), and that cite mathematical oncology* (right) for all focus journals between 1961 and 2024. (c) The results in (b) are shown for one focus journal at a time in pie charts. (d) The results in (b) are shown over time. For each citation, it is the time (year) that the citation was made that is shown in one of the plots. (e) Annual counts for articles that are cited by (top) and that cite (bottom) mathematical oncology*. Counts for both discipline-classified and unclassified journals are shown. The notation mathematical oncology* refers to the studied dataset

As a preparatory step, we map out inter-citation patterns between our five mathematical biology focus journals. The citation matrix in Figure 2a demonstrates that mathematical oncology articles published in JTB, MB, and MBE have the most citations from themselves, whereas mathematical oncology articles in BMB and JMB – the flagship journals of the Society for Mathematical Biology and the European Society for Mathematical and Theoretical Biology, respectively – obtain most of their citations from JTB. This demonstrates the societies’ intradisciplinary reach, although it is partly explained by JTB being the focus journal with the largest number of articles. Indeed, the effect of journal size on self-citations has been observed in scientometric studies (Taşkin et al. 2021). To mitigate this effect, we include an alternative version of the citation matrix in the Supplementary Material (S5), using normalisation by the number of mathematical biology* articles in each respective journal. The normalised matrix shows that all focus journals, except JTB, account for the highest fraction of citations within themselves, indicating strong internal citation patterns even after adjusting for journal size in the mathematical biology* dataset.

Next, to identify the disciplines that *influence* and *are influenced by* mathematical oncology, we extend our scope to study all articles that *are cited by* and/or *cite* articles from our mathematical oncology* dataset. We classify these articles into disciplines based on their journals, following the scheme outlined in Section 2.6. For each discipline, journals that most frequently are cited by and cite the mathematical oncology* dataset are listed in the Supplementary Material (S5). With our scheme, we are able to classify 81% of the articles that are cited by mathematical oncology*, and 82% that cite mathematical oncology*.

The spectra of disciplines that are cited by, and cite, the full mathematical oncology* dataset are shown in Figure 2b through the citation-V. The figure reflects that the vast majority of articles cited by mathematical oncology* belong to life science journals (58.2%). Thereafter, the focus journals (11.3%) and multidisciplinary journals (9.9%) are the most cited. Notably, only 3.7% of the cited articles are published in mathematics journals. When we instead examine which articles cite mathematical oncology*, the disciplinary composition is more diffused, meaning citations come from a broader and more evenly distributed range of fields. Most strikingly, the percentage of articles from life science journals has been reduced to 27.8%, while articles from mathematics journals have increased to 12.7%. In between, articles from the focus journals account for 18.0% of the citations. Thus, to paraphrase Reed’s article *Mathematical biology is good for mathematics* (2015), our quantitative results suggest that mathematical oncology is good for, or at least cited by, mathematics.

When breaking this result down to a journal level, we see that citing trends differ among our focus journals (Figure 2c). While life science articles account for the majority of citations in all focus journals, ranging from 49.0% in JMB to 60.8% in JTB, mathematics articles account for 8.9% of in JMB, but less than half of that in all other journals. Even on a journal level, the diffusive effect of mathematical oncology is clear as the composition of disciplines that cite mathematical oncology* articles is significantly more evenly distributed than the composition of disciplines cited by mathematical oncology*.

The last result raises the question: *is this diffusive effect something that has emerged over time?* To approach this research question, we plot the proportion of articles that are cited by, and cite, mathematical oncology* over time in Figure 2d. The plot demonstrates a clear shift over time; while life science disciplines dominated both the “cited by” and “citing” categories in the 1960s, the representation of mathematics has, overall, grown over time and has had a steady presence since the early 2000s. The representation of focus journals in both categories has remained largely consistent since the 1990s, as have the related disciplinary categories “mathematics and life sciences” and “STEM and life sciences”. These results interestingly contradict the theory that research fields tend to become increasingly internalised and interact less with external disciplines as they mature (Singh et al. 2022), as is further elaborated on in the Discussion (Section 4). To allow for relating the percentage-based results reported in this section to absolute numbers, we plot the total number of mathematical oncology* references and citations in Figure 2e. The corresponding results for each individual focus journal are available in the Supplementary Material (S5).

### On internationality in mathematical oncology

**Fig. 3:**
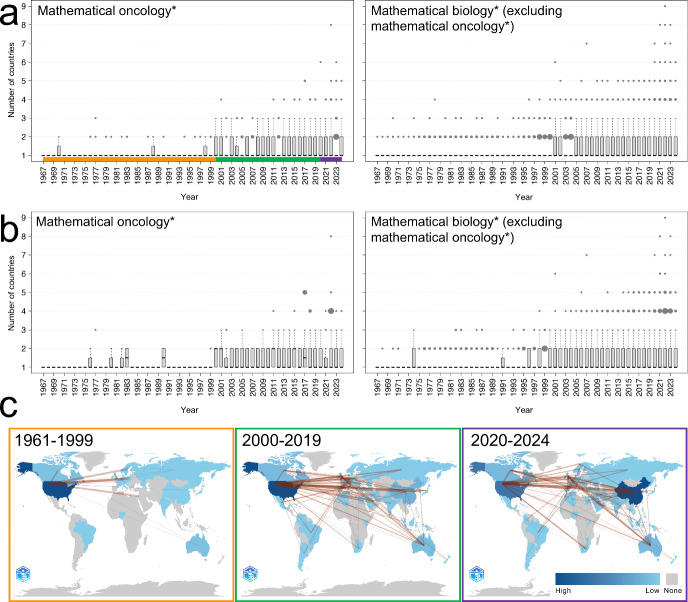
**Internationality in mathematical oncology** The box plots show the number of unique author affiliation-countries per article over time for (a) all articles in the datasets, and (b) articles with above-average citations published that year. In the box plots, black lines indicate the medians; boxes show the interquartile ranges (IQR); whiskers extend to the smallest and largest data points within 1.5$\times$IQR from the box edges; and black dots represent outliers, with dot size proportional to their frequency. (c) The heatmaps show country-wise contributions (number of articles) to the mathematical oncology* dataset for three different time eras. Red lines demonstrate pairwise collaborations between countries, where the line thickness is proportional to collaboration frequency. Heatmap colours and line thicknesses are normalised for each time period. The notations mathematical oncology* and biology* refer to the studied datasets

Motivated by research question R2, we next set out to study how international mathematical oncology is by analysing author affiliation countries in article metadata. The box plots in Figure 3 show how many affiliation countries are represented per mathematical oncology* article, and how these numbers have changed over time since 1967, the first year with available affiliation data. To situate our results in a broader context, we also generate the corresponding box plots for the mathematical biology* (excluding mathematical oncology*) dataset. For both datasets, we further extract the articles with above-average (per publication year) citations to investigate whether there is any link between an article’s country representation and its citation count. We do this by comparing medians (Q2) and the lower and upper quartiles (Q1 and Q3) across datasets. Note that these quartile values take half-integer values in the event of ties.

We find that the mathematical oncology* dataset demonstrates a slight correlation between country counts and citation counts: in 7 out of 58 years, articles with above-average citation counts have a higher median number of affiliation countries than the complete dataset, and in 11 of 58 years they have a higher Q3 value. The reverse is true in only 0 and 4 years, respectively. We further observe a similar correlation in the mathematical biology* (excluding mathematical oncology*) dataset: in 7 out of 58 years, articles with above-average citation counts have a higher Q3 value than all articles combined in the dataset, with the reverse never being true.

While the Q1 and Q2 values have remained 1 over time, for all articles in both datasets, Figure 3a demonstrates a slow increase in country count over time. This is reflected by the strong Pearson correlation coefficients for Q3 between 1961 and 2024 (0.7162 and 0.8321 for the mathematical oncology* and mathematical biology* (excluding mathematical oncology*) datasets respectively) (Mukaka 2012). Moreover, the most notable shifts in country affiliation counts are observed around the years 2000 and 2020, and we therefore split the studied time period into three eras: the pre-millennia era (1961-1999), the early 21st century (2000-2019), and the 2020s (2020-2024). We speculate that the last split is related to the COVID-19 pandemic which notably impacted the culture of STEM research (Heo et al. 2022). Pearson correlation coefficient values for Q1, Q2, and Q3, across all sub-datasets and time eras, are provided in the Supplementary Material (S6).

For each era, countries that contribute to the mathematical oncology* dataset are highlighted on world maps (Figure 3c), with colours representing the number of article contributions per country. The maps are generated in bibliometrix (Aria and Cuccurullo 2017). For each article, every unique author affiliation contributes one count to the country heatmap, and each distinct country pair contributes one count to the collaboration edges. For example, if an article has two authors from different institutions within the same country, that country contributes two counts. Conversely, if an article includes at least one author from country A and one from country B, it is counted as a single A-and-B collaboration, regardless of the number of affiliations within each country. Both country-wise article counts and pairwise collaboration frequencies are available in tabulated form in the Supplementary Material (S6).

Together, the three maps indicate a geographical decentralisation of mathematical oncology* over time, following general scientific globalisation trends (Dong et al. 2017). It is interesting to note that when new countries appear on the map in the last two eras, they typically do so as connected nodes to previously engaged countries. This pattern can, for instance, be observed across three continents in the early 21st century map, by panning from left to right and noting the addition of Argentina, South Africa, and New Zealand. Although these results demonstrate correlation, not causation, they still signify the importance of enabling research connections to support globalisation. This is in line with previous studies on sustainable partnerships between the Global North and South for research (Kunert et al. 2020) and education (Mahdjoub et al. 2023) purposes. As such, organisations aiming to support the globalisation of mathematical oncology can use the findings reported in this section in two important ways: first, to identify underrepresented countries; and second, to motivate financial and infrastructural support for international collaboration networks.

### On collaboration in mathematical oncology

**Fig. 4:**
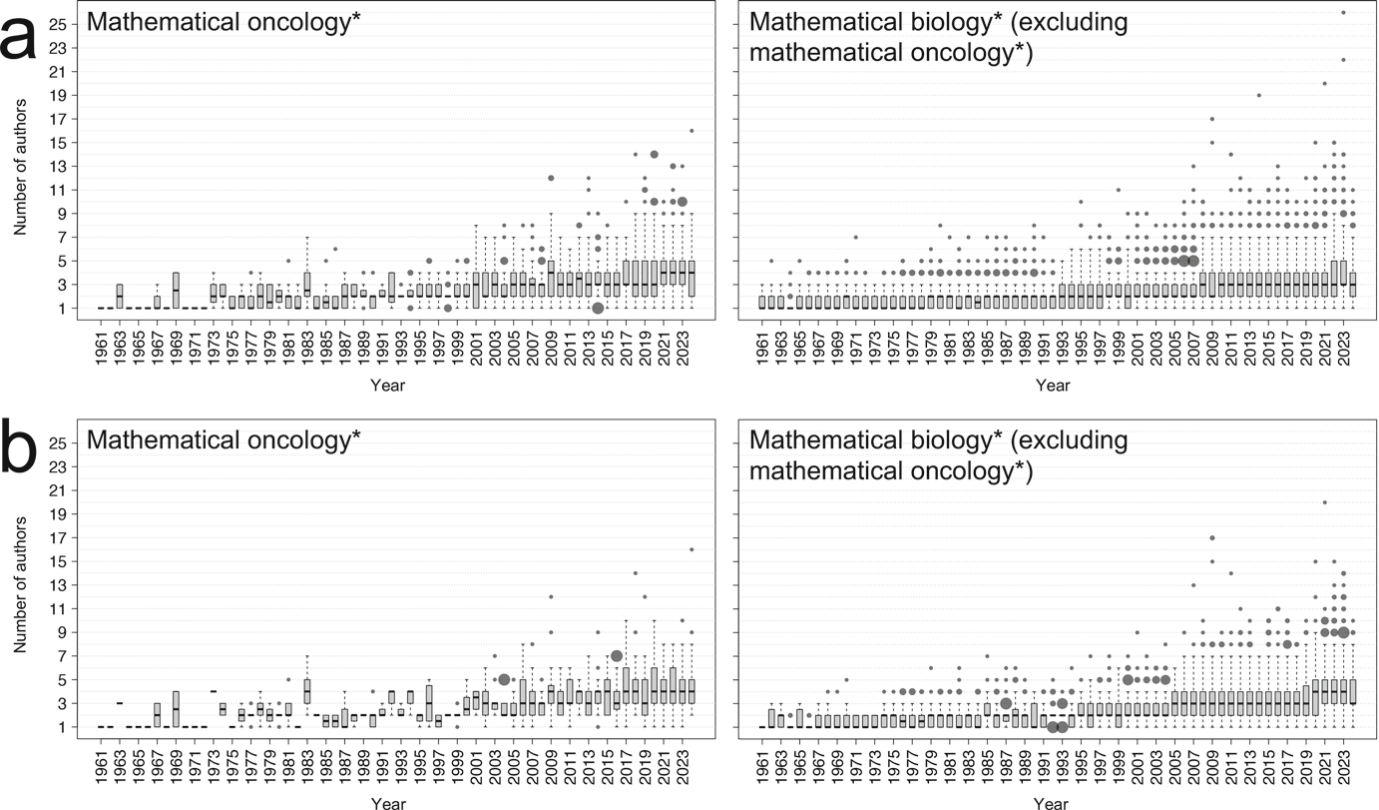
**Collaboration in mathematical oncology.** The box plots show the number of authors per article over time for (a) all articles in the datasets and (b) articles with above-average citations published that year. In the box plots, black lines indicate the medians; boxes show the interquartile ranges (IQR); whiskers extend to the smallest and largest data points within 1.5$\times$IQR from the box edges; and black dots represent outliers, with sizes proportional to their frequency. The notations mathematical oncology* and biology*, refer to the studied datasets

Continuing the discussion on collaborations and turning to research question R3, we now analyse team sizes in mathematical oncology* studies, again building on author metadata. This is particularly interesting as the field overlaps both mathematics, a discipline historically dominated by small teams and single-author articles, and biomedicine, which typically involves larger collaborative teams (Porter and Rafols 2009).

Taking a similar approach as in the previous section, we use box plots to visualise how the number of authors per article has changed over time in Figure 4a. The figure reveals a slow and steady increase over time in the number of authors per article in both the mathematical oncology* and mathematical biology* (excluding mathematical oncology*) datasets, as is quantified by strong Pearson correlation coefficient values over 0.8 for Q1, Q2, and Q3 during 1961-2024 for both datasets (Mukaka 2012). These results follow general trends for life science publications (Rao et al. 2022) and science more broadly (Larivière et al. 2015; Fortunato et al. 2018). Comparing the two plots in Figure 4a we also see that, since 2017, mathematical oncology* articles have generally had slightly larger team sizes (in terms of interquartile ranges) than mathematical biology* articles as a whole. We conjecture that this may be related to the broader trend that, within the biomedical literature, publications directly or closely related to clinical implementation have experienced the highest increases in team sizes since the turn of the millennium (Jakab et al. 2024).

We next focus on citation counts again and see that, for the mathematical oncology* articles, the median number of authors is higher in articles with above-average citation counts compared to the complete dataset in 22 out of 64 years, and lower in only 3. A similar pattern appears for the lower and upper quartiles, with Q1 and Q3 values being higher for above-average cited articles in 21 and 16 years, respectively, and lower in only 4 and 13 years. These results signify that articles with above-average citation counts are generally associated with larger sized teams.

In a broader scientific context, bigger team sizes are generally correlated with higher impact through citations, an outcome that is not merely a consequence of self-citations, but may also be a product of the pooled knowledge that goes into a study when researchers work together, especially across disciplines (Larivière et al. 2015). In mathematical oncology studies, where it is not uncommon for mathematicians, data scientists, biologists, and clinicians to collaborate, it is thus natural to reason that increased team sizes would come with a citation-count benefit. A similar, but slightly weaker, correlation between team sizes and citation counts is identified for the mathematical biology* (excluding mathematical oncology*) articles. This is shown in Figure 4, where the median number of authors is higher for articles with above-average citations compared to all articles in the dataset in 13 out of 64 years, with the reverse being true in only 3 years. Similarly, Q1 and Q3 are higher for above-average cited articles in 10 and 12 years, respectively, and lower in only 1 and 4 years. All Pearson correlation coefficient values for Q1, Q2, and Q3, across each dataset and time era, are provided in the Supplementary Material (S7).

### On trending topics in mathematical oncology

**Fig. 5:**
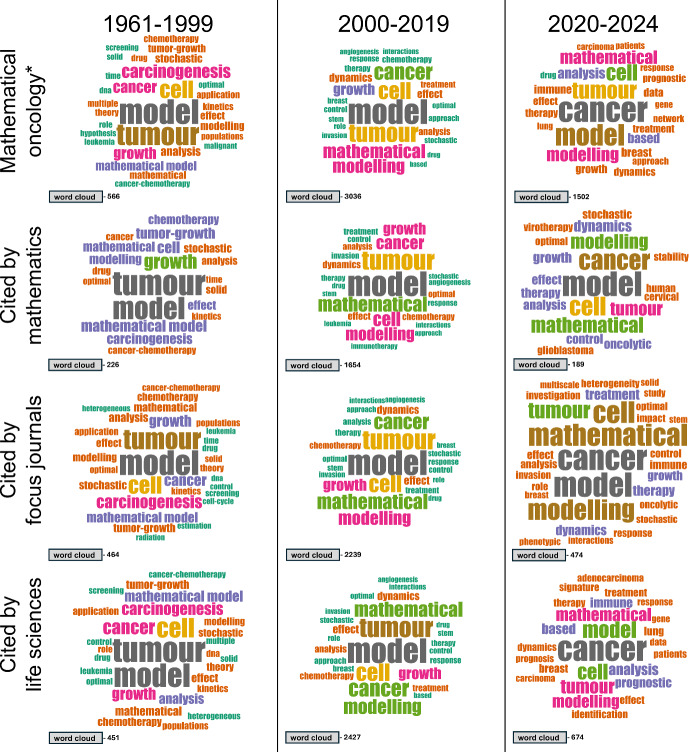
**Trending topics in mathematical oncology analysed through title word frequency.** In each subplot, the bar chart shows the frequency of the collective word cloud terms. The word clouds contain the 25 most frequent terms with sizes proportional to their frequency. Data for all mathematical oncology* abstracts, per time period, are shown in the top row. Data for articles with at least one citation in mathematics, focus, and life science journals are shown in the three rows below, respectively

**Fig. 6:**
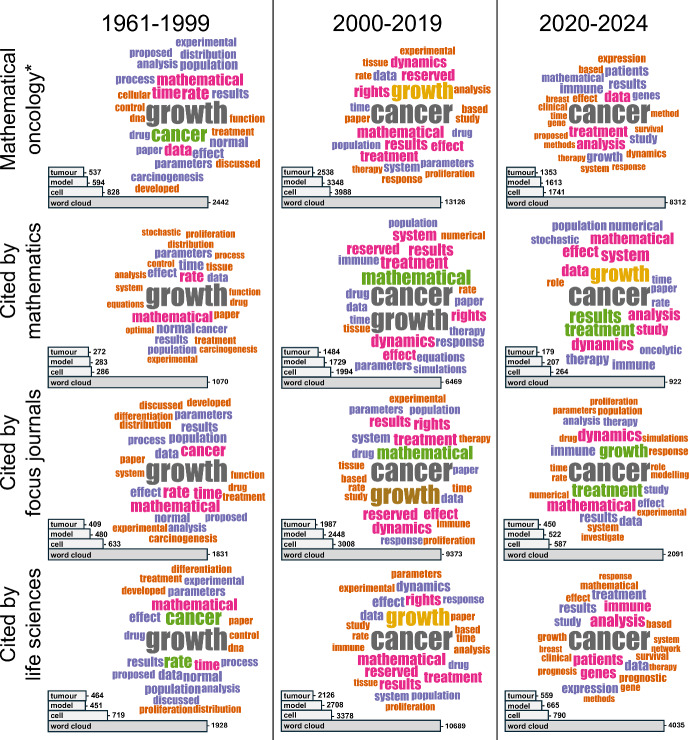
**Trending topics in mathematical oncology analysed through abstract word frequency.** In each subplot, the bar charts show the frequency of the terms tumour, model, cell, and the collective word cloud terms. The word clouds contain the 25 most frequent terms after similarity pruning (removing tumour, model, cell) with sizes proportional to their frequency. Data for all mathematical oncology* abstracts, per time period, are shown in the top row. Data for articles with at least one citation in mathematics, focus, and life science journals are shown in the three rows below, respectively

We now set out to investigate research question R4 by identifying trending research topics in mathematical oncology and how these have varied over time. We do this via word clouds, which can be used to flexibly visualise term frequency in bibliometric datasets without predefined categories or keywords. The word clouds in the top rows of Figures 5 and 6, respectively, show the frequency of words in the titles and abstracts of our mathematical oncology* dataset over the three time periods defined in Section 3.2. The word cloud data are generated in bibliometrix, which automatically filters out common stopwords (e.g., “and", “the", “or") from the analysis. Additionally, to reduce redundant counting of terms, we created our own list of synonyms which is provided in the Supplementary Material (S8). Tabulated word cloud data, and corresponding histograms, are also available in the Supplementary Material (S8).

Extending our prior investigation into which disciplines consume (are influenced by) mathematical oncology (Section 3.1), Figures 5 and 6 contain word clouds for all articles cited by (at least one article in) three journal categories: our focus journals, mathematics, and life science journals. Together, these word clouds highlight similarities and differences between time periods and consumer groups. We first note that the words cell, model, and tumour have consistently been in the top three abstract words (and the top six title words) for all time periods and consumer groups, indicating stability of the field. To prevent these high-frequency words from dominating the abstract word clouds and obfuscating information (Figure 6), we display them through bars instead of words.

Moreover, the word clouds reveal shifts in trending topics that are homogeneous across consumer groups; angiogenesis is present in all title clouds in the early 2000s but not elsewhere, and immune appears in all consumer-group abstract clouds for the last two time eras. We conjecture that these observations are related: the latter reflects the series of immunotherapy breakthroughs that have transformed cancer treatments since the 2000s (Dobosz and Dzieciatkowski 2019), and the former that anti-angiogenic agents were first FDA approved in 2004 and are now predominantly used and studied in combination with other therapies, such as immunotherapy (Ansari 2022). Another word that is present in all word clouds for the abstracts is data, but only in the 2020s does the word feature in the title clouds – and then only for the complete and life science consumer groups. This suggests that, although data have always been an integral part of the field, it is only recently that authors have promoted (or been awarded for promoting) the use of data in article titles, which is presumably linked to the fact that cancer research is becoming increasingly data-driven (Jiang et al. 2022).

The finding that life science, but not mathematics, journals cite articles with data in the title indicates a split in interests between the two consumer groups. Meanwhile, the word equations is present only in the cited-by-mathematics abstract word clouds. Similarly, the related words numerical and simulation only appear in the word clouds for the mathematics and mathematical biology (focus journal) groups. In line with this observed split, Fawcett and Higginson (2012) argue that heavy use of equations impedes communication in biology articles, whereas Chitnis and Smith (2012) rebut this statement by claiming that it is mathematical illiteracy that is hindering the use of equations in biology. Regardless of which stance one takes in this debate, our analysis reveals distinct differences between articles cited by mathematics versus life science journals, with those cited by mathematical biology lying somewhere in between.

Further, the title word clouds for the complete dataset and the cited-by-life sciences suggest an increased specificity towards cancer types over time, as words like breast and lung increase in frequency. We also infer from the title data that articles cited by life science journals have recently become more focused on clinical applications, as the words clinical, patients, and prognostic first appear in the 2020s, where the last word also indicates a move towards using mathematical oncology for predictions – a task where machine learning models commonly outperform mechanistic ones, given sufficient data (Baker et al. 2018). Related trends towards data, machine learning and the encompassing field of AI have been observed in other areas of applied mathematics (Li et al. 2025) and science overall (Hajkowicz et al. 2023) through bibliometric studies.

## Discussion

Our bibliometric research reveals that, over the past seven decades, mathematical oncology has undergone a significant evolution marked by increased interactions with other disciplines, geographical expansion, larger research teams, and greater diversity in research topics. To further characterise the role that mathematical oncology plays in the larger knowledge system, we here draw on Kwon et al.’s framework (2017) which classifies citation flows and, by extension, interdisciplinary knowledge flows, into three types: aggregating, bridging, and diffusing. Aggregating articles cite two or more distinct source disciplines, whereas bridging articles cite one discipline and are cited by another, and diffusing articles are cited by multiple disciplines. From our data analysis, we find that mathematical oncology performs all three of these roles, but not symmetrically. It does not typically aggregate mathematics and oncology directly; instead it primarily draws on knowledge from the life sciences and adjacent disciplines that combine the life sciences with other areas of STEM, often integrating quantitative approaches into biological contexts. Furthermore, our observed diffusive and bridging citation patterns support Reed’s hypothesis that mathematical biology (and its subfields) benefits both mathematics and biology (Reed 2015).

These knowledge flow patterns contrast with those described by Singh et al. (2022), who find that scientific fields typically become more internally focused as they mature, citing external disciplines less frequently. Their findings align with Eddy’s (2005) notion of ‘antedisciplinary’ science. Using molecular biology as an example, Eddy argues that early work in new disciplines emerges outside existing disciplines and later consolidates into its own self-contained identity. Mathematical oncology, on the other hand, appears to mature without such disciplinary closure. Rather than narrowing its scope, the field has become more interdisciplinary over time; continually drawing on and contributing to both mathematics and the life sciences. Mathematical oncology has, of course, developed a disciplinary identity of its own, as reflected by dedicated research communities, events, and education tracks, but the field continues to thrive through active engagement with both mathematics and the life sciences.

While citation flows illustrate disciplinary connections, they reveal little about *how* researchers engage with mathematical oncology. To this end, word clouds provide important complementary insight into the nature of mathematical oncology research that different disciplines interact with. As discussed in the previous section, mathematical oncology* articles published in the last five years that are cited by life science journals increasingly emphasise the use of data in their titles, and tend to focus on clinical applications and prediction. This may reflect the broader rise in data availability within cancer research (Jiang et al. 2022), but also signals a shift in emphasis; while data has long been part of abstracts in the field, its recent prominence in titles suggests changing expectations around what makes an article valuable (or citable) in the life sciences.

In contrast, articles cited by mathematics journals more often feature terms such as equations, numerical, and simulation. While this observation, as discussed in the previous section, indeed reflects the debate put forward by Fawcett and Higginson (2012) and Chitnis and Smith (2012) centered around the presentation of equations in theoretical biology papers, it also touches upon an even older discussion in mathematical biology on the values and trade-offs in modelling (Levins 1966). Levins argued that, when modelling biological systems, no model can simultaneously maximise generality, realism, and precision. Our word cloud analysis indicates that, over the last years, the life science community increasingly value models focusing on precision, whereas the mathematics community seem to focus on models that Levins would categorise as emphasising generality.

These patterns may point to the beginning of a broader disciplinary divergence, where mathematical oncology is increasingly split between data-driven, clinically oriented work and more abstract, mathematically focused modelling. While we cannot determine an article’s modelling goals from keyword analysis alone, the observed shifts in terminology raise important questions about which kinds of models different communities find useful or persuasive. What makes a mathematical model of a biological system *good* – mechanistic insight, predictive accuracy, or clinical relevance? Or; are mathematical models that prioritise mechanistic understanding of biological processes becoming less relevant in a research landscape increasingly dominated by machine learning models focused on prediction? Baker et al. (2018) explore this tension and propose hybrid approaches that combine mechanistic modelling with machine learning techniques as a way forward. Complementing this discussion, Gyllingberg et al. (2023) argue that for mathematical models to remain relevant to the life sciences, the field of mathematical biology must avoid an overemphasis on mathematical rigour and instead adopt a more pluralistic, biologically grounded modelling practice.

Although our bibliometric research offers a window into how different communities engage with mathematical oncology, our results are based solely on citation patterns and keyword matches in article metadata. In future work, further information on interdisciplinarity in mathematical oncology could be gathered from author affiliations and training backgrounds, in line with previous work by Abramo et al. (2012). To better segment our findings, future studies could also apply established interdisciplinarity metrics, such as the Rao–Stirling index (Stirling 2007), to more precisely quantify how interdisciplinary different journals are. In our word cloud analysis, we focused on articles cited by different communities, but future work could also examine which papers draw more heavily on mathematics, the life sciences, or both, and compare these patterns to a mathematical biology baseline. In addition, a more in-depth analysis of which modelling approaches are valued by different communities would offer important insight into the epistemic priorities shaping the field. To extract such information, approaches based on large language models (LLMs) are likely to outperform basic keyword-based methods. LLMs could also be used in pipelines for extracting and analysing data on commonly studied cancer types and interventions from article metadata and, alternatively, full article texts. The potential of LLMs is illustrated by their recent applications in bibliometric research; from automating keyword extraction that reflect scientific content (Mansour et al. 2025) and categorising scientific texts (Shahi and Hummel 2025), to evaluating research works (Evans et al. 2024). With this rise of LLMs, it becomes increasingly important to ground bibliometric studies in domain knowledge of the studied field, theoretical frameworks, and transparent methodologies.

## Conclusion and outlook

In this study, we use bibliometric methods to get a bird’s-eye view of mathematical oncology research practices. We anticipate that our study will encourage further bibliometric research on mathematical oncology and, more broadly, mathematical biology, that quantitatively probes questions that are typically studied qualitatively. For instance, a growing collection of literature reviews and perspective articles highlights the importance and potential of mathematical oncology, see e.g., Altrock et al. (2015), Rockne (2019), and Kuznetsov et al. (2021). In support of these works, our bibliometric study adds data-driven, quantitative evidence of the field’s interdisciplinary reach. Further, our study provides insights into how author teams, citation flows, and research topics have changed in the field since the 1960s. For us working in mathematical oncology, we can use these insights to inform our research, teaching, organisation, and communication practices. For the good of cancer research, mathematics, and our research community.

## Supplementary Information

Below is the link to the electronic supplementary material.

Supplementary file 1 (pdf 454 KB)

## Data Availability

The code used in this study is available on the public GitHub repository https://github.com/KiraPugh/Bibliometric_Study_Mathematical_Oncology. Data included in this study are mainly derived from the Web of Science (Clarivate); and partly from Scopus (Elsevier).
