# Supplementary material for: A bibliometric study on mathematical oncology: interdisciplinarity, internationality, collaboration and trending topics
Source: Bull Math Biol. 2025 Nov 4;87(12):174. doi: 10.1007/s11538-025-01544-9 (PMC12583306; doi:10.1007/s11538-025-01544-9)
Supplement: Supplementary file 1 — (pdf 454 KB) [file 11538_2025_1544_MOESM1_ESM.pdf]

Supplementary material to A bibliometric study on mathematical oncology: interdisciplinarity, internationality, collaboration and trending topics

Kira Pugh⊛, Linnéa Gyllingberg, Stanislav Stratiev, Sara Hamis⊛.

⊛ **Corresponding authors**: kira.pugh@it.uu.se, sara.hamis@it.uu.se.

Contents

S1 Journal selection **2**

S2 Journal classification **4**

S3 Journal coverage on the WoSCC database **5**

S4 Article classification **6**

S5 Supporting information on interdisciplinarity **7**

S6 Supporting information on internationality **10**

S7 Supporting information on collaboration **14**

S8 Supporting information on trending topics **14**

S9 Code files **17**

S1 Journal selection

We start our journal selection procedure by searching the Web of Science Core Collection (WoSCC) database for articles that match the topic search (TS) keywords below.

TS = ((cancer* OR tumour* OR tumor* OR oncolog* OR oncogen* OR carcinogen*) AND mathematic*).

Journal-level frequencies of keyword matches are available in Table S1.1, for all journals with 100 or more matches. The table also includes journal classification codes based on the Quacquarelli Symonds (QS) system, identifying four journals categorised as mathematics and life sciences journals (underlined) as well as two journals categorised as journals with mathematics, life sciences, and other content (italicised). To supplement the journal classification results, we list the self-authored journal descriptions in Table S1.2, where we make judgements about which journals focus on mathematical biology (underlined). Based on the information in the two tables we classify the following five as our mathematical biology focus journals (in alphabetical order):

1. the Bulletin of Mathematical Biology (BMB),
2. the Journal of Mathematical Biology (JMB),
3. the Journal of Theoretical Biology (JTB),
4. Mathematical Biosciences (MB),
5. Mathematical Biosciences and Engineering (MBE).

Table S1.1: **Journals with more that 100 articles on the WoSCC database that match the TS keywords in the title, abstract, and/or author keywords.** Journals belonging to the QS subject area *mathematics*; the QS broad faculty area *life sciences and medicine*; and any other QS subject or faculty areas are check-marked.

Journal	Keyword matching article count	Mathematics subject area	Life sciences and medicine faculty area	Other
Journal of Theoretical Biology	455	✓	✓	
PLOS One	313			✓
Bulletin of Mathematical Biology	276	✓	✓	✓
Scientific Reports	244			✓
Cancer Research	239		✓	
PLOS Computational Biology	236	✓	✓	✓
Mathematical Biosciences	222	✓	✓	
Mathematical Biosciences and Engineering	160	✓	✓	
Medical Physics	160		✓	
Physics in Medicine and Biology	155		✓	
Proceedings of SPIE	140			
Cancers	126		✓	
Proceedings of the National Academy of Sciences of the United States of America	121			✓
International Journal of Radiation Oncology Biology Physics	120		✓	
Journal of Mathematical Biology	119	✓	✓	

Table S1.2: **Self-authored descriptions of the journals listed in Table S1.** The descriptions are pasted from the journals' websites. Journals that explicitly highlight both mathematics and biology are underlined.

Journal name	Summary of aims and scope
Journal of Theoretical Biology	The Journal of Theoretical Biology is the leading forum for theoretical perspectives that give insight into biological processes. Acceptable papers are those that bear significant importance on the biology per se being presented, and not on the mathematical analysis.
PLOS One	PLOS ONE welcomes original research submissions from the natural sciences, medical research, engineering, as well as the related social sciences and humanities.
Bulletin of Mathematical Biology	As the official journal of the Society for Mathematical Biology, this journal shares research at the biology-mathematics interface. It publishes original research, mathematical biology education, reviews, commentaries, and perspectives.
Scientific Reports	We publish original research from all areas of the natural sciences, psychology, medicine and engineering.
Cancer Research	Cancer Research seeks manuscripts that offer conceptual or technological advances leading to basic and translational insights into cancer biology. Manuscripts that focus on convergence science, the bridging of two or more distinct areas of cancer research, are of particular interest.
PLOS Computational Biology	By making connections through the application of computational methods among disparate areas of biology, PLOS Computational Biology provides substantial new insight into living systems at all scales, from the nano to the macro, and across multiple disciplines, from molecular science, neuroscience and physiology to ecology and population biology.
Mathematical Biosciences	Mathematical Biosciences publishes work providing new concepts or new understanding of biological systems using mathematical models, or methodological articles likely to find application to multiple biological systems.
Mathematical Biosciences and Engineering	MBE focuses on new developments in the fast-growing fields of mathematical biosciences and engineering. Areas covered include most areas of mathematical and computational biology, medicine and engineering with an emphasis on integrative and interdisciplinary research bridging mathematics, biology and engineering.
Medical Physics	Medical Physics publishes high-quality articles across radiological imaging, nuclear medicine, radiation oncology, and clinical radiation effects. Topics include new methodologies, imaging or treatment device development, artificial intelligence applications, and clinical or theoretical studies. Radiological imaging covers modalities like CT, MRI, and ultrasound, with a focus on computational analysis such as radiomics and CAD.
Physics in Medicine and Biology	The novel and impactful development and application of theoretical, computational and experimental physics to medicine, physiology and biology.
Proceedings of SPIE	Conference where proceedings are published in the SPIE Digital Library which features more than 600,000 publications in optics and photonics.
Cancers	Cancers (ISSN 2072-6694) is an international, peer-reviewed open access journal on oncology.
Proceedings of the National Academy of Sciences of the United States of America	PNAS publishes exceptional research across all areas of the biological, physical, and social sciences. Innovation often happens at the margins of disciplines, and we are especially interested in research that crosses disciplinary bounds, answers questions with broad scientific impact, or breaks new ground.
International Journal of Radiation Oncology Biology Physics	International Journal of Radiation Oncology - Biology - Physics (IJROBP), known in the field as the Red Journal, publishes original laboratory and clinical investigations related to radiation oncology, radiation biology, medical physics, and both education and health policy as it relates to the field.
Journal of Mathematical Biology	The Journal of Mathematical Biology (JOMB) focuses on scientific advancements in mathematical modelling and analysis of biological systems.

Links to journal descriptions are listed below and were accessed on 2025-06-21.

- Journal of Theoretical Biology: `https://www.sciencedirect.com/journal/journal-of-theoretical-biology/about/aims-and-scope`
- PLOS One: `https://journals.plos.org/plosone/s/journal-information`
- Bulletin of Mathematical Biology: `https://link.springer.com/journal/11538/aims-and-scope`
- Scientific Reports: `https://www.nature.com/srep/about`
- Cancer Research: `https://aacrjournals.org/cancerres/pages/about`
- PLOS Computational Biology: `https://journals.plos.org/ploscompbiol/s/journal-information`
- Mathematical Biosciences: `https://www.sciencedirect.com/journal/mathematical-biosciences/about/aims-and-scope`
- Mathematical Biosciences and Engineering: `https://www.aimspress.com/mbe/news/solo-detail/aimandscope`
- Medical Physics: `https://aapm.onlinelibrary.wiley.com/hub/journal/24734209/overview`
- Physics in Medicine and Biology: `https://publishingsupport.iopscience.iop.org/journals/physics-in-medicine-biology/about-physics-medicine-biology`
- Proceedings of SPIE: `https://www.spiedigitallibrary.org/conference-proceedings-of-spie`
- Cancers: `https://www.mdpi.com/journal/cancers/about`
- Proceedings of the National Academy of Sciences of the United States of America: `https://www.pnas.org/author-center`
- International Journal of Radiation Oncology Biology Physics: `https://www.sciencedirect.com/journal/international-journal-of-radiation-oncology-biology-physics/about/aims-and-scope`
- Journal of Mathematical Biology: `https://link.springer.com/journal/285/aims-and-scope`

S2 Journal classification

In this study we classify journals as belonging to one of seven categories:

1. *focus journal,*
2. *mathematics,*
3. *life sciences,*
4. *multidisciplinary,*
5. *mathematics and life sciences,*
6. *STEM and life sciences,*
7. *other.*

From journal titles in our metadata, we use a previously developed lookup table (Himmelstein, 2024) to map journal titles to one or more ASJC codes (Elsevier, 2025) via Scopus IDs. Next, we use the QS scheme (QS Quacquarelli Symonds, 2025) which classifies each ASJC code as belonging to one "broad faculty area" and one "subject area". From a combination of Scopus IDs, ASJC codes, QS broad faculty areas, and QS subject areas we customise the journal classification used in this study, following Algorithm 1.

Algorithm 1 Journal classification.

Input: Journal name
if Journal **is** focus journal **then**
  Classify as *focus journal*
else if subject area **is only** mathematics **then**
  Classify as *mathematics*
else if broad faculty area **is only** life sciences and medicine **then**
  Classify as *life sciences*
else if ASJC code **is only** multidisciplinary **then**
  Classify as *multidisciplinary*
else if subject area **includes only** mathematics **and** a subject area from the broad faculty area life sciences and medicine **then**
  Classify as *mathematics and life sciences*
else if broad faculty area **includes only** (life sciences and medicine) **and** (natural sciences **and/or** engineering and technology) **then**
  Classify as *STEM and life sciences*
else
  Classify as *other*
end if
Output: Journal class

Codes to map journals to our customised classes are available on the study's GitHub repository.

S3 Journal coverage on the WoSCC database

In Table S3.1 we list the coverage of our focus journals on the WoSCC database. The coverage is approximated as

$$\frac{\#\text{articles on the WoSCC}}{\#\text{articles on journal website}} \times 100\%,$$

where editorials and indexing publications have been omitted. The JTB coverage of over 100% results from retracted publication and addendums being included on the WoSCC.

Table S3.1: **The WoSCC coverage of the focus journals.**

**Journal**	**#of articles on the WoSCC**	**#of articles on journal website**	**WoSCC coverage**
Bull. Math. Biol.	4102	4355	94.19%
J. Math. Biol.	3689	3744	98.53%
J. Theor. Biol.	16759	16728	100.19%
Math. Biosci.	5037	5329	94.00%
Math. Biosci. Eng	3946	4188	94.22%

S4 Article classification

In Figure 1 of the main manuscript, we illustrate the procedure of classifying whether an article belongs to the mathematical oncology* or mathematical biology* (excluding mathematical oncology*) dataset. This classification is based on keyword matches, and the choice of keywords are based on their predictive performance. The list of words used in candidate word groups (WG) 1-3 are shown in Figure S4.1, where the words in WG2 include all cancer types that are listed by the National Cancer Institute (2025), Cancer Research UK (2024), the American Cancer Society (2025), Wikipedia (2025), and WebMD (2025); and have subsequently been confirmed as cancer types by the authors. To assess the accuracy resulting from classification with WG1, WG2\WG1, WG3\(WG1∪WG2), authors KP and SH reviewed 100 randomly selected articles (20 per focus journal) predicted to be true (in mathematical oncology*), and 100 predicted to be false. From the confusion matrix in Table S4.1, we compute that the accuracy is 0.985 for WG1, 0.925 for WG1\WG2, and 0.505 for WG3\(WG1∪WG2). From these results, we decide to use WG1∪WG2 in the cancer-article classification.

Word group 1

- Cancer
- Tumour
- Tumor
- Oncolog
- Oncogen
- Carcinogen

Word group 2

- Melanoma
- Leukemia
- Leukaemia
- Carcinoma
- Lymphoma
- Blastoma
- Glioma
- Sarcoma
- Myeloma
- Adamantinoma
- Adenoma
- Astrocytoma
- Birt-hogg-dubé
- Histiocytoma
- Carcinoid
- Craniopharyngioma
- Chordoma
- Ependymoma
- Epithelioid hemangioendothelioma
- Glucagonoma
- Insulioma
- Keratoacanthoma
- Linitis plastica
- Meningioma
- Mesenchymoma
- Mesothelioma
- Multiple endocrine neoplasia
- Mycosis fungoide
- Myelodysplastic
- Myelodysplasia
- Myeloproliferative
- Myelofibrosis
- Neurofibroma
- Somatostatinoma
- Teratoma
- Thymoma
- VIPoma
- Waldenstrom macroglobulinaemia
- Waldenstrom macroglobulinemia
- Polycythaemia vera
- Polycythemia vera
- Oseudomyxoma peritonei
- Schwannoma
- Seminoma
- Sézary
- Essential thrombocythaemia
- Essential thrombocythemia
- Ganglioneuroma
- Gastrinoma
- Gestational trophoblastic
- Pheochromocytoma
- Plasmacytoma
- Recurrent respiratory papillomatosis
- Acoustic neuroma
- Mastocytoma
- Pineocytoma
- Gangliocytoma

Word group 3

- Chemotherapy
- Radiotherapy
- Radiation therapy
- Ionizing radiation
- Ionising radiation
- Immunotherapy
- Targeted therapy
- Stem cell therapy
- Hyperplasia
- Atypia
- Metaplasia
- Dysplasia
- Neoplasia
- Neoplasm
- Metasta
- Neoplastic
- Angiogenesis
- Biopsy
- Malignan

Figure S4.1: **Analysis of cancer-class-keywords.** A list of all cancer-class-keywords included in the candidate keyword groups WG1, WG2, and WG3.

	True Positive	True Negative
Predicted Positive	WG1: 99<br>WG2\WG1: 88<br>WG3\(WG1∪WG2): 4	WG1: 1<br>WG2\WG1: 12<br>WG3\(WG1∪WG2): 96
Predicted Negative	WG1: 2<br>WG2\WG1: 3<br>WG3\(WG1∪WG2): 3	WG1: 98<br>WG2\WG1: 97<br>WG3\(WG1∪WG2): 97

Table S4.1: **Confusion matrix for candidate cancer-class-keywords.**

S5 Supporting information on interdisciplinarity

Citing journals (columns); Cited journals (rows)

Cited journals	Bull. Math. Biol. (3630)	J. Math. Biol. (2967)	J. Theor. Biol. (13432)	Math. Biosci. (4778)	Math. Biosci. Eng. (3881)
Bull. Math. Biol. (337)	0.1468	0.0772	0.0485	0.0525	0.0618
J. Math. Biol. (178)	0.0636	0.0863	0.0288	0.0213	0.0348
J. Theor. Biol. (814)	0.1603	0.1207	0.1230	0.0682	0.0773
Math. Biosci. (379)	0.0678	0.0455	0.0248	0.1153	0.0366
Math. Biosci. Eng. (442)	0.0168	0.0148	0.0062	0.0098	0.0523

Figure S5.1: **Normalised citation matrix.** The citation matrix shows how often mathematical oncology* articles in the focus journals (rows) are cited by other focus journals (columns). To account for differences in journal size, the citation counts are normalised by the number of articles in the citing journals (within the mathematical biology* dataset). The total number of articles per journal are shown in parentheses. The maximum number of citations per row are red and underlined.

Table S5.1: **Top ten journals in each journal category that are cited by and cite mathematical oncology*.** Total article counts are shown in parentheses.

Category	Journal name (cited by mathematical oncology*)	Journal name (citing mathematical oncology*)
Mathematics and life sciences	Biometrics (148)	Computational and Mathematical Methods in Medicine (300)
Mathematics and life sciences	Computational and Mathematical Methods in Medicine (133)	Theoretical Biology and Medical Modelling (206)
Mathematics and life sciences	Biology Direct (133)	Biosystems (194)
Mathematics and life sciences	Theoretical Biology and Medical Modelling (114)	Biology Direct (103)
Mathematics and life sciences	Biosystems (85)	IET Systems Biology (82)
Mathematics and life sciences	Statistics in Medicine (71)	Statistics in Medicine (70)
Mathematics and life sciences	CPT: Pharmacometrics & Systems Pharmacology (60)	Biometrics (65)
Mathematics and life sciences	IET Systems Biology (40)	CPT-Pharmacometrics & Systems Pharmacology (62)
Mathematics and life sciences	Statistical Methods in Medical Research (23)	Current Bioinformatics (60)
Mathematics and life sciences	Cellular and Molecular Bioengineering (19)	Cellular and Molecular Bioengineering (52)
Mathematics	Mathematical Models & Methods in Applied Sciences (393)	Discrete and Continuous Dynamical Systems-Series B (641)
Mathematics	Discrete and Continuous Dynamical Systems-Series B (307)	Mathematical Models & Methods in Applied Sciences (489)
Mathematics	SIAM Journal on Applied Mathematics (297)	Mathematical Modelling of Natural Phenomena (465)
Mathematics	Mathematical Modelling of Natural Phenomena (173)	Communications in Nonlinear Science and Numerical Simulation (314)

Table S5.1 continued.

Mathematics	SIAM Review (129)	International Journal of Biomathematics (244)
Mathematics	Journal of Mathematical Analysis and Applications (114)	Journal of Mathematical Analysis and Applications (212)
Mathematics	Applied Mathematics Letters (108)	Applied Mathematical Modelling (208)
Mathematics	European Journal of Applied Mathematics (93)	SIAM Journal on Applied Mathematics (202)
Mathematics	SIAM Journal on Mathematical Analysis (78)	Journal of Differential Equations (163)
Mathematics	Studies in Applied Mathematics (70)	AIMS Mathematics (143)
Life sciences	Cancer Research (3371)	Cancer Research (371)
Life sciences	Cell (1233)	Cancers (351)
Life sciences	Nature Reviews Cancer (1032)	Frontiers in Oncology (298)
Life sciences	British Journal of Cancer (859)	Physics in Medicine and Biology (207)
Life sciences	Biophysical Journal (749)	Biophysical Journal (190)
Life sciences	Journal of Biological Chemistry (695)	Wiley Interdisciplinary Reviews-Systems Biology and Medicine (171)
Life sciences	Blood (626)	Cell Proliferation (154)
Life sciences	Clinical Cancer Research (558)	Physical Biology (149)
Life sciences	New England Journal of Medicine (514)	Frontiers in Immunology (142)
Life sciences	Journal of Immunology (494)	Seminars in Cancer Biology (126)
Multidisciplinary	Proceedings of the National Academy of Sciences of the United States of America (2280)	PLOS One (1134)
Multidisciplinary	Nature (2170)	Scientific Reports (692)
Multidisciplinary	Science (1490)	Proceedings of the National Academy of Sciences of the United States of America (191)
Multidisciplinary	PLOS One (1182)	Royal Society Open Science (166)
Multidisciplinary	Scientific Reports (465)	iScience (112)
Multidisciplinary	Scientific American (62)	Heliyon (76)
Multidisciplinary	Science Advances (36)	Nature (26)
Multidisciplinary	Royal Society Open Science (29)	Science Advances (17)
Multidisciplinary	iScience (14)	omptes Rendus de L'Academie Bulgare des Sciences (15)
Multidisciplinary	Chinese Science Bulletin (8)	Science (14)
STEM and life sciences	PLOS Computational Biology (751)	PLOS Computational Biology (879)
STEM and life sciences	Nature Communications (346)	Journal of the Royal Society Interface (354)
STEM and life sciences	Radiation Research (344)	Journal of Biological Systems (238)
STEM and life sciences	Journal of the Royal Society Interface (314)	Computers in Biology and Medicine (226)
STEM and life sciences	International Journal of Radiation Oncology Biology Physics (294)	International Journal of Molecular Sciences (226)
STEM and life sciences	International Journal of Molecular Sciences (179)	Biomechanics and Modeling in Mechanobiology (174)
STEM and life sciences	Nature Biotechnology (168)	BMC Systems Biology (156)
STEM and life sciences	Risk Analysis (157)	Biomedical Signal Processing and Control (121)

Table S5.1 continued.

STEM and life sciences	BMC Systems Biology (142)	International Journal for Numerical Methods in Biomedical Engineering (119)
STEM and life sciences	Proceedings of the Royal Society B-Biological Sciences (140)	NPJ Systems Biology and Spplications (114)
Other	Mathematical and Computer Modelling (523)	Physical Review E (488)
Other	Physical Review E (513)	Mathematical and Computer Modelling (413)
Other	Physical Review Letters (250)	Chaos Solitons & Fractals (392)
Other	Journal of Computational Physics (186)	Mathematical Methods in the Applied Sciences (348)
Other	Development (130)	Mathematics (321)
Other	Nonlinearity (97)	Nonlinear Analysis-Real World Applications (305)
Other	Journal of Chemical Physics (96)	Computers & Mathematics with Applications (198)
Other	Journal of the American Statistical Association (83)	Nonlinearity (197)
Other	IEEE Access (79)	European Physical Journal Plus (150)
Other	Multiscale Modeling & Simulation (79)	IEEE Access (119)

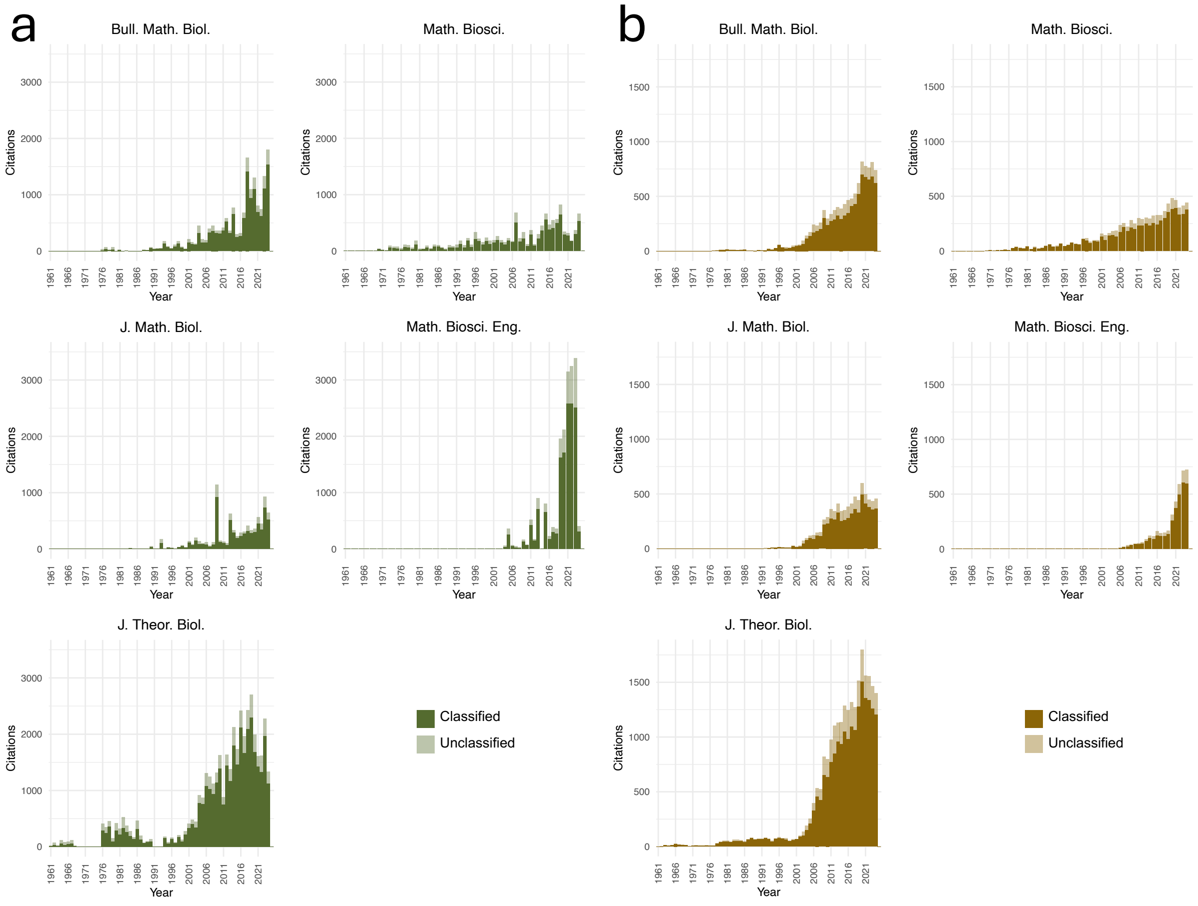

Figure S5.2: **Annual counts for articles that are cited by (left) and that cite (bottom) mathematical oncology*.** Counts for both discipline-classified and unclassified journals are shown.

S6 Supporting information on internationality

We have affiliation-county metadata for 94.5% of the articles in the mathematical oncology* dataset, and 87.7% of the mathematical biology* (excluding mathematical oncology*) dataset . Of the 2150 mathematical oncology* articles, 118 articles have unspecified country affiliations. Among these, 101 articles have no affiliation information available when article metadata is downloaded from WoSCC (9 from BMB, 3 from JMB, 49 from JTB, and 40 from MB), with only 2 published in or after 2000. The remaining 17 articles, all published before 2000, contain unrecognised country names. Therefore, per time era the country coverage of the mathematical oncology* dataset is 64.5% (1961-1999), 99.8% (2000-2019), and 100% (2020-2024).

Figure S6.1 displays the Pearson correlation coefficients for Q1, Q2, and Q3 over time (1961-2024), as well as for the three time eras we consider in our study, corresponding to the box plots in Figure 3. For the three time eras, country-wise article counts are listed in Tables S6.1-S6.3; and pairwise collaboration counts are listed in Tables S6.4-S6.6.

a

Mathematical oncology*

	Q1	Q2	Q3
1961-2024	-	-	0.7162
1961-1999	-	-	0.0775
2000-2019	-	-	0.3179
2020-2024	-	-	-0.3536

Mathematical biology* (excluding mathematical oncology*)

	Q1	Q2	Q3
1961-2024	-	-	0.8321
1961-1999	-	-	-
2000-2019	-	-	0.5221
2020-2024	-	-	-

b

Mathematical oncology*

	Q1	Q2	Q3
1961-2024	-	0.1783	0.7645
1961-1999	-	0.0934	0.0178
2000-2019	-	-0.1563	0.2984
2020-2024	-	-	0.3536

Mathematical biology* (excluding mathematical oncology*)

	Q1	Q2	Q3
1961-2024	-	-	0.8142
1961-1999	-	-	0.2478
2000-2019	-	-	-
2020-2024	-	-	-

Figure S6.1: **Pearson Correlation Coefficients for number of unique author affiliation-countries per article over time.** Values were computed for the lower quartile (Q1), median (Q2), and upper quartile (Q3) over different time periods for (a) all articles in the datasets and (b) those with above-average citations in the corresponding year. Values in red, orange, and green denote strong ($0.7 < r \leq 1$), moderate ($0.5 \leq r < 0.7$), and no or weak ($r < 0.5$) Pearson correlation coefficients r, respectively. When a tabulated quantity shows no variation across years, the Pearson correlation coefficient is not defined, as is indicated by '–'.

Table S6.1: **Number of articles (N) in the mathematical oncology* dataset between 1961 and 1999.**

**Country**	**N**	**Country**	**N**	**Country**	**N**	**Country**	**N**
USA	218	Sweden	7	Russia	4	Croatia	1
UK	42	Italy	6	India	3	Denmark	1
Australia	18	Israel	5	Poland	3	Nigeria	1
Canada	18	Brazil	4	Austria	2	Norway	1
Germany	15	Finland	4	Czechia	2	Slovakia	1
Japan	15	France	4	Bulgaria	1	Sri Lanka	1
Belgium	8	Netherlands	4	China	1	*Unspecified*	116

Table S6.2: **Number of articles (N) in the mathematical oncology* dataset between 2000 and 2019.**

Country	N	Country	N	Country	N	Country	N
USA	1126	India	30	Austria	9	Indonesia	3
United UK	395	Netherlands	28	Norway	9	Kuwait	3
China	229	Portugal	23	Turkey	9	Qatar	3
France	217	Russia	23	Romania	8	Algeria	2
Italy	168	Cuba	16	Finland	7	Bulgaria	2
Canada	133	Pakistan	16	Saudi Arabia	6	Morocco	2
Germany	116	Argentina	15	Hungary	5	Bangladesh	1
Spain	82	Belgium	15	Singapore	5	Botswana	1
Australia	81	Switzerland	15	Egypt	4	Colombia	1
Iran	61	Sweden	12	Serbia	4	Czechia	1
Japan	52	Ireland	11	Slovakia	4	Kenya	1
Poland	38	South Korea	11	Tunisia	4	Lebanon	1
Israel	37	Denmark	10	Venezuela	4	Malaysia	1
Brazil	32	Mexico	10	Chile	3	Eswatini	1
New Zealand	32	South Africa	10	Greece	3	*Unspecified*	2

Table S6.3: **Number of articles (N) in the mathematical oncology* dataset between 2020 and 2024.**

Country	N	Country	N	Country	N	Country	N
China	533	Turkey	10	Cyprus	4	Tunisia	2
USA	406	Chile	9	Iraq	4	Azerbaijan	1
UK	116	Brazil	8	Morocco	4	Belgium	1
France	71	Indonesia	8	Hungary	3	Cuba	1
Italy	70	Netherlands	8	Ireland	3	Kazakhstan	1
Germany	52	New Zealand	8	Israel	3	Luxembourg	1
Canada	51	Portugal	8	Lesotho	3	Nigeria	1
Australia	49	South Korea	8	Poland	3	Oman	1
Spain	44	Greece	7	Slovakia	3	Philippines	1
India	39	Malaysia	7	Uganda	3	Senegal	1
Sweden	27	Russia	7	Algeria	2	Serbia	1
Norway	22	Switzerland	7	Argentina	2	Singapore	1
Iran	20	Colombia	6	Bangladesh	2	Ukraine	1
Pakistan	18	Denmark	6	Benin	2	Yemen	1
Finland	17	Egypt	6	Lebanon	2	Zimbabwe	1
Saudi Arabia	16	Japan	6	Romania	2	*Unspecified*	0
Mexico	12	Vietnam	6	Rwanda	2		
South Africa	11	Austria	4	Thailand	2		

Table S6.4: **Number of shared articles (N) in the mathematical oncology* dataset between 1961 and 1999.**

Country 1	Country 2	N	Country 1	Country 2	N	Country 1	Country 2	N
USA	Canada	2	Finland	Russia	1	USA	Italy	1
USA	Israel	2	Germany	Russia	1	USA	Japan	1
USA	Poland	2	Sweden	Norway	1	USA	Russia	1
USA	Sweden	2	USA	Australia	1			

Table S6.5: **Number of shared articles (N) in the mathematical oncology* dataset between 2000 and 2019.**

Country 1	Country 2	N	Country 1	Country 2	N	Country 1	Country 2	N
USA	UK	41	Poland	Israel	2	Italy	Venezuela	1
USA	China	34	Spain	Brazil	2	Japan	Austria	1
USA	France	18	UK	Austria	2	Japan	Ireland	1
USA	Germany	16	UK	Portugal	2	Netherlands	Austria	1
USA	Canada	15	USA	Argentina	2	Netherlands	Finland	1
UK	Australia	13	USA	Pakistan	2	Netherlands	Sweden	1
UK	France	13	USA	Singapore	2	New Zealand	Singapore	1
UK	Germany	13	USA	Switzerland	2	Pakistan	Korea	1
UK	Canada	10	Argentina	Belgium	1	Pakistan	Saudi Arabia	1
Italy	Spain	9	Australia	India	1	Poland	Egypt	1
USA	Italy	9	Australia	Mexico	1	Poland	South Africa	1
USA	Japan	9	Australia	Netherlands	1	Poland	Switzerland	1
UK	Italy	7	Australia	New Zealand	1	Portugal	Argentina	1
France	Italy	6	Belgium	Austria	1	Portugal	Belgium	1
USA	Australia	6	Belgium	Greece	1	Portugal	Cuba	1
USA	India	6	Belgium	Ireland	1	Portugal	Mexico	1
USA	Poland	6	Brazil	Cuba	1	Portugal	South Africa	1
USA	Russia	6	Brazil	Mexico	1	Russia	Mexico	1
USA	Spain	6	Brazil	Portugal	1	Saudi Arabia	Egypt	1
Germany	Russia	5	Brazil	Switzerland	1	South Africa	Bulgaria	1
Italy	Germany	5	Canada	Denmark	1	South Africa	Swaziland	1
UK	Ireland	5	Canada	Ireland	1	Spain	Argentina	1
UK	Spain	5	Canada	Israel	1	Spain	Australia	1
USA	Israel	5	Canada	Japan	1	Spain	Bulgaria	1
USA	Netherlands	5	Canada	Netherlands	1	Spain	Cuba	1
Australia	Korea	4	Canada	UAE	1	Spain	Norway	1
Canada	Australia	4	Chile	Colombia	1	Spain	Poland	1
China	Japan	4	China	Austria	1	Spain	Russia	1
France	Germany	4	China	Egypt	1	Spain	Serbia	1
UK	China	4	China	Germany	1	Spain	South Africa	1
USA	Brazil	4	China	Italy	1	Spain	Switzerland	1
USA	Denmark	4	China	Kenya	1	Sweden	Finland	1
USA	Iran	4	China	New Zealand	1	UK	Argentina	1
USA	Korea	4	China	Spain	1	UK	Bangladesh	1
USA	Saudi Arabia	4	Cuba	Mexico	1	UK	Belgium	1
USA	South Africa	4	France	Argentina	1	UK	Brazil	1
Australia	Poland	3	France	Brazil	1	UK	Finland	1
France	Israel	3	France	Canada	1	UK	Greece	1
France	Spain	3	France	Lebanon	1	UK	Japan	1
France	Switzerland	3	France	Netherlands	1	UK	Netherlands	1
Germany	Hungary	3	France	Poland	1	UK	New Zealand	1
Germany	Poland	3	France	Russia	1	UK	Poland	1
Germany	Spain	3	France	Saudi Arabia	1	UK	Qatar	1
Spain	Mexico	3	France	South Africa	1	UK	Russia	1
Spain	Portugal	3	Germany	Australia	1	UK	Saudi Arabia	1
UK	Israel	3	Germany	Austria	1	UK	South Africa	1
USA	Austria	3	Germany	Brazil	1	UK	Switzerland	1
USA	Portugal	3	Germany	Bulgaria	1	UK	Turkey	1
USA	Qatar	3	Germany	Greece	1	UK	UAE	1
Australia	Romania	2	Germany	New Zealand	1	USA	Bangladesh	1
Canada	Germany	2	Germany	Portugal	1	USA	Botswana	1
China	Australia	2	Germany	South Africa	1	USA	Egypt	1
China	Canada	2	Iran	Sweden	1	USA	Hungary	1
China	Saudi Arabia	2	Ireland	Austria	1	USA	Indonesia	1
China	Singapore	2	Israel	Mexico	1	USA	Malaysia	1
France	Australia	2	Israel	Sweden	1	USA	Mexico	1
France	Austria	2	Italy	Argentina	1	USA	New Zealand	1
France	Japan	2	Italy	Australia	1	USA	Romania	1
France	Qatar	2	Italy	Austria	1	USA	Turkey	1
Germany	Switzerland	2	Italy	Brazil	1	USA	UAE	1
Italy	Canada	2	Italy	Cuba	1	USA	Venezuela	1
Italy	Mexico	2	Italy	New Zealand	1			
Italy	Netherlands	2	Italy	Switzerland	1			

Table S6.6: **Number of shared articles (N) in the mathematical oncology* dataset between 2020 and 2024.**

Country 1	Country 2	N	Country 1	Country 2	N	Country 1	Country 2	N
USA	UK	15	Canada	Saudi Arabia	1	Italy	Turkey	1
China	USA	11	Canada	South Africa	1	Korea	Hungary	1
USA	Canada	10	Canada	Thailand	1	Korea	Lebanon	1
UK	France	9	Canada	Turkey	1	Korea	Slovakia	1
UK	Italy	8	Canada	Zimbabwe	1	Korea	UAE	1
UK	Australia	7	Chile	Brazil	1	Morocco	Tunisia	1
China	Australia	5	Chile	Luxembourg	1	Netherlands	Belgium	1
China	UK	5	China	Algeria	1	Netherlands	Israel	1
France	Italy	5	China	Azerbaijan	1	Norway	Austria	1
Italy	Spain	5	China	Bangladesh	1	Norway	Korea	1
China	Pakistan	4	China	France	1	Norway	Lebanon	1
Pakistan	Saudi Arabia	4	China	Nigeria	1	Norway	Portugal	1
UK	Spain	4	China	Philippines	1	Norway	UAE	1
UK	Switzerland	4	China	Rwanda	1	Pakistan	Azerbaijan	1
USA	France	4	China	Saudi Arabia	1	Pakistan	Egypt	1
USA	Italy	4	China	Singapore	1	Pakistan	Hungary	1
USA	Norway	4	China	South Africa	1	Pakistan	Korea	1
USA	Spain	4	China	Spain	1	Pakistan	Slovakia	1
Australia	Saudi Arabia	3	China	Thailand	1	Pakistan	Thailand	1
Canada	Iran	3	China	Yemen	1	Russia	Benin	1
China	Canada	3	Colombia	Zimbabwe	1	Russia	Morocco	1
Saudi Arabia	Malaysia	3	Egypt	Algeria	1	Rwanda	Nigeria	1
South Africa	Lesotho	3	Egypt	Yemen	1	Saudi Arabia	Egypt	1
UK	Germany	3	Finland	Netherlands	1	Saudi Arabia	Hungary	1
UK	Saudi Arabia	3	France	Chile	1	Saudi Arabia	Indonesia	1
USA	Australia	3	France	Cyprus	1	Saudi Arabia	Iraq	1
USA	Lesotho	3	France	Luxembourg	1	Saudi Arabia	Korea	1
USA	South Africa	3	France	Netherlands	1	Saudi Arabia	Slovakia	1
USA	Switzerland	3	France	Poland	1	South Africa	Algeria	1
Australia	India	2	France	Senegal	1	South Africa	Colombia	1
Australia	Korea	2	France	South Africa	1	South Africa	Egypt	1
Australia	Malaysia	2	France	Sweden	1	South Africa	Yemen	1
China	Egypt	2	France	Tunisia	1	South Africa	Zimbabwe	1
China	Germany	2	France	Zimbabwe	1	Spain	Mexico	1
China	Iraq	2	Germany	Canada	1	Spain	Serbia	1
China	Italy	2	Germany	Denmark	1	Sweden	Austria	1
China	Japan	2	Germany	Iran	1	Sweden	Finland	1
China	Norway	2	Germany	Israel	1	Sweden	Norway	1
Finland	Austria	2	Germany	Netherlands	1	Sweden	Russia	1
France	Canada	2	Germany	New Zealand	1	Thailand	Azerbaijan	1
France	Colombia	2	Germany	Pakistan	1	Turkey	Japan	1
France	Morocco	2	Germany	Spain	1	Turkey	Morocco	1
France	Russia	2	Germany	Turkey	1	UAE	Lebanon	1
France	Spain	2	Hungary	Slovakia	1	UK	Brazil	1
Italy	Australia	2	India	Korea	1	UK	Canada	1
Italy	Brazil	2	India	Lebanon	1	UK	Cyprus	1
Italy	Canada	2	India	Malaysia	1	UK	Finland	1
Norway	Finland	2	India	Norway	1	UK	Greece	1
Pakistan	Iraq	2	India	Pakistan	1	UK	India	1
Spain	Brazil	2	India	Saudi Arabia	1	UK	Nigeria	1
Sweden	Morocco	2	India	UAE	1	UK	Norway	1
UK	Ireland	2	Indonesia	Malaysia	1	UK	Oman	1
UK	Netherlands	2	Indonesia	Ukraine	1	UK	Rwanda	1
USA	Germany	2	Iran	Egypt	1	UK	Sweden	1
USA	Netherlands	2	Iran	Finland	1	USA	Algeria	1
USA	Russia	2	Iran	Pakistan	1	USA	Brazil	1
USA	Sweden	2	Iran	Saudi Arabia	1	USA	Finland	1
Algeria	Thailand	1	Iraq	Azerbaijan	1	USA	Greece	1
Algeria	Yemen	1	Iraq	Thailand	1	USA	Kazakhstan	1
Australia	Indonesia	1	Israel	Belgium	1	USA	Korea	1
Australia	New Zealand	1	Italy	Austria	1	USA	Mexico	1
Canada	Australia	1	Italy	Azerbaijan	1	USA	Morocco	1
Canada	Azerbaijan	1	Italy	India	1	USA	New Zealand	1
Canada	Colombia	1	Italy	Iraq	1	USA	Pakistan	1
Canada	Iraq	1	Italy	Morocco	1	USA	Poland	1
Canada	Korea	1	Italy	New Zealand	1	USA	Senegal	1
Canada	Malaysia	1	Italy	Pakistan	1	USA	Thailand	1
Canada	New Zealand	1	Italy	Sweden	1			
Canada	Pakistan	1	Italy	Thailand	1			

S7 Supporting information on collaboration

Figure S7.1 displays the Pearson correlation coefficients for Q1, Q2, and Q3 for all box plots in Figure 4 in the main manuscript.

a

Mathematical oncology*

	Q1	Q2	Q3
1961-2024	0.8151	0.8618	0.8407
1961-1999	0.6984	0.5516	0.5029
2000-2019	0.4628	0.4432	0.6162
2020-2024	0	0.7071	-

Mathematical biology* (excluding mathematical oncology*)

	Q1	Q2	Q3
1961-2024	0.8425	0.9139	0.9129
1961-1999	0.3822	0.8089	0.6846
2000-2019	0.3780	0.8453	0.8496
2020-2024	0.3536	-	0.2887

b

Mathematical oncology*

	Q1	Q2	Q3
1961-2024	0.6073	0.7201	0.7702
1961-1999	0.2556	0.3466	0.3464
2000-2019	0.3860	0.5380	0.6333
2020-2024	-	-	-0.5773

Mathematical biology* (excluding mathematical oncology*)

	Q1	Q2	Q3
1961-2024	0.8501	0.8917	0.9093
1961-1999	0.5227	0.6701	0.6149
2000-2019	-	0.7509	0.7959
2020-2024	0.7071	-0.7071	-

Figure S7.1: **Pearson Correlation Coefficients for the number of authors per article over time.** Values were computed for the lower quartile (Q1), median (Q2), and upper quartile (Q3) over different time periods for (a) all articles in the datasets and (b) those with above-average citations in the corresponding year. Values in red, orange, and green denote strong ($0.7 < r \leq 1$), moderate ($0.5 \leq r < 0.7$), and no or weak ($r < 0.5$) Pearson correlation coefficients r, respectively. When a tabulated quantity shows no variation across years, the Pearson correlation coefficient is not defined, as is indicated by '–'.

S8 Supporting information on trending topics

The frequency of the word clouds presented in Figures 5 and 6 in the main manuscript are generated with the R-package biblioshiny (Aria and Cuccurullo, 2017). Synonyms are combined in the word clouds as shown in Table S8.1 and for the abstract word clouds we remove the words `cell`, `model`, and `tumour` according to a similarity pruning. For each word cloud, the 25 most frequent words are included; as long as they appear at least 5 times. In cases where multiple words (with frequency of at least 5) are tied for 25th place, they are all included. Complementary to the word clouds, bar charts displaying the frequency of the top 25 words in article titles and abstracts are shown in Figures S8.1 and S8.2 respectively. In the study's GitHub repository, word cloud frequencies are available in numeric form in the file `word_cloud_data.csv`.

Table S8.1: **A list of synonyms used when generating the word clouds.**

Term	Synonyms
Mathematical model	Mathematical-model; Mathematical models; Mathematical-models
Tumour	Tumor; Tumours; Tumors
Cell	Cells
Modelling	Modeling
Model	Models
Effect	Effects

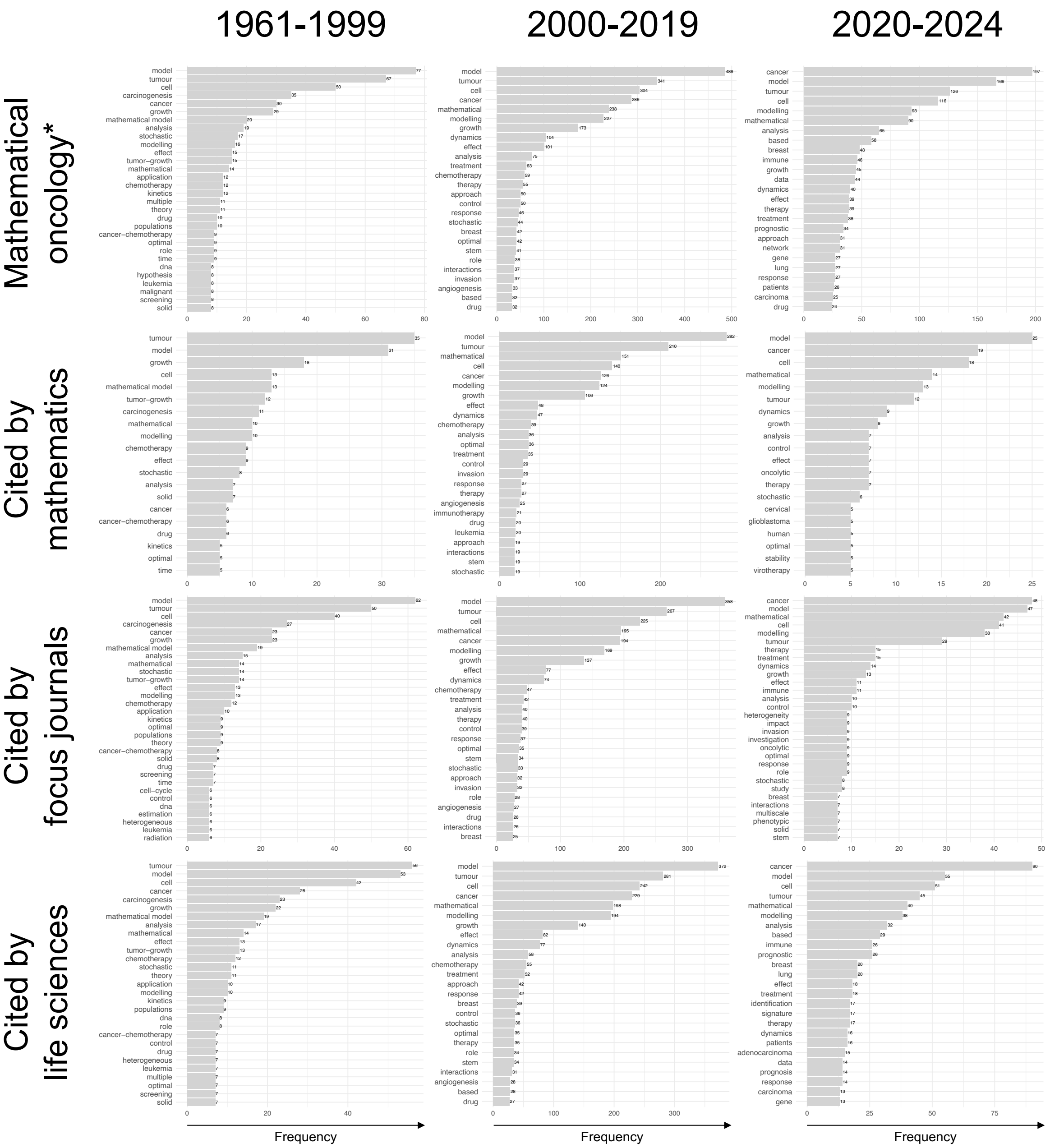

Figure S8.1: **Trending topics in mathematical oncology analysed through title word frequency.** The bar charts contain the 25 most frequent terms, with frequencies next to the bars. Data for all mathematical oncology* titles, per time period, are shown in the top row. Data for articles with at least one citation in mathematics, focus, and life science journals are shown in the three rows below, respectively.

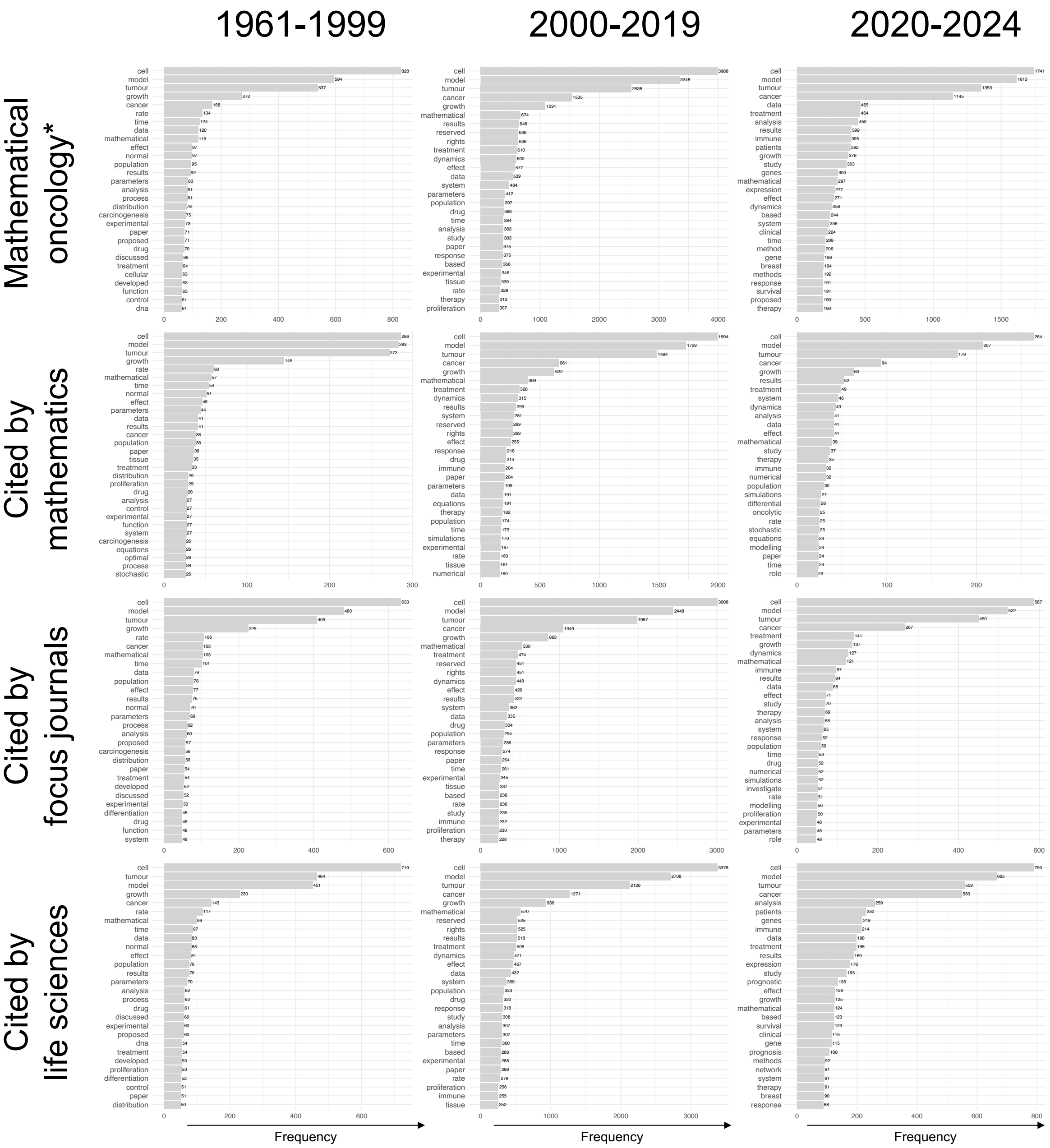

Figure S8.2: **Trending topics in mathematical oncology analysed through abstract word frequency.** The bar charts contain the 25 most frequent terms, with frequencies next to the bars. Data for all mathematical oncology* abstract, per time period, are shown in the top row. Data for articles with at least one citation in mathematics, focus, and life science journals are shown in the three rows below, respectively.

S9 Code files

The code files used in this study are available on the public GitHub repository https://github.com/KiraPugh/Bibliometric_Study_Mathematical_Oncology. Instructions on how to run the code files are available in the repository's README file.

References

American Cancer Society (2025). *Cancer Types.* Accessed: 2025-06-12. URL: https://www.cancer.org/cancer/types.html.

Aria, M. and C. Cuccurullo (2017). "Bibliometrix: An R-tool for comprehensive science mapping analysis", *Journal of Informetrics* 11.4, pp. 959–975. DOI: 10.1016/j.joi.2017.08.007.

Cancer Research UK (2024). *Your cancer type.* Accessed: 2025-06-12. URL: https://www.cancerresearchuk.org/about-cancer/type.

Elsevier (2025). *All Science Journal Classifications: ASJC Codes.* Accessed: 2025-06-09. URL: https://support.qs.com/hc/en-gb/articles/4406036892562-All-Science-Journal-Classifications-ASJC-Codes.

Himmelstein, D. S. (2024). *scopus.* Accessed: 2025-06-21. URL: https://github.com/dhimmel/scopus.

National Cancer Institute (2025). *Cancer Types.* Accessed: 2025-06-12. URL: https://www.cancer.gov/types.

QS Quacquarelli Symonds (2025). *QS World University Rankings by Subject.* Accessed: 2025-06-09. URL: https://support.qs.com/hc/en-gb/articles/4410488025106-QS-World-University-Rankings-by-Subject.

WebMD (2025). *Cancer A-Z.* Accessed: 2025-06-12. URL: https://www.webmd.com/cancer/cancer-a-to-z.

Wikipedia (2025). *List of cancer types.* Accessed: 2025-06-12. URL: https://en.wikipedia.org/wiki/List_of_cancer_types.
